# Effects of a Novel Protease on Growth Performance, Nutrient Digestibility and Intestinal Health in Weaned Piglets

**DOI:** 10.3390/ani12202803

**Published:** 2022-10-17

**Authors:** Qingqing Zhu, Yuxin Wang, Yanjie Liu, Bing Yu, Jun He, Ping Zheng, Xiangbing Mao, Zhiqing Huang, Junqiu Luo, Yuheng Luo, Hui Yan, Jie Yu

**Affiliations:** 1 Key Laboratory for Animal Disease-Resistance Nutrition of Sichuan Province, Animal Nutrition Institute, Sichuan Agricultural University, Chengdu 611130, China; 2 Jinan Bestzyme Bio-Engineering Co., Ltd., Jinan 250101, China; 3 Sichuan Tequ Agriculture and Animal Husbandry Technology Group Co., Ltd., Chengdu 610207, China

**Keywords:** protease, weaning piglets, digestibility, intestinal health

## Abstract

**Simple Summary:**

A novel type of protease with high stability, high resistance to acid and a wider range of enzyme cutting sites has been developed recently. This experiment was conducted to investigate the effects of the novel protease in weaned piglets. Our results indicate that dietary protease supplementation promotes nutrient absorption, improves small intestine morphology and enhances digestive enzyme activities of weaned piglets.

**Abstract:**

This experiment was conducted to evaluate the effects of different protease levels on performance, diarrhea rate, nutrient digestibility, blood metabolites, digestive enzyme activities, and intestinal health of weaned piglets. A total of 96 weaned piglets (Duroc × Landrace × Yorkshire, 28 d of age, initial average BW = 6.55 ± 0.17 kg) were divided into four groups (4 pigs/pen and 6 replicates/group) according to a completely random block design. Piglets were fed different levels of protease (0, 150, 300 or 600 mg/kg of diet) for 28 d. The results showed that the addition of protease had no significant effect on the growth performance of weaned piglets (*p* > 0.05), and the addition of 300 mg/kg protease significantly increased the apparent total intestinal digestibility (ATTD) of nutrients and the apparent ileal digestibility (AID) of amino acids in weaned piglets (*p* < 0.05), while the addition of 150 mg/kg and 600 mg/kg protease had no significant effect on the digestibility (*p* > 0.05). The nutrient digestibility of dry matter (DM), organic matter (OM), crude protein (CP) and total energy (GE) showed a trend of increasing and then decreasing with increasing protease concentration (*p* < 0.05). Adding 300 and 600 mg/kg protease significantly decreased serum ALB/GLO levels (*p* < 0.05) and duodenal pH (*p* < 0.05) and increased duodenal villus height (*p* < 0.05). The addition of protease significantly increased jejunal trypsin and chymotrypsin activities (*p* < 0.01) and duodenal and jejunal mucosal tight junction proteins in piglets. The mRNA expression levels of ZO-1 and CLAUDIN-1 in the duodenum together with ZO-1 and OCCLUDIN in the jejunum of piglets in the 300 and 600 mg/kg protease supplementation groups were significantly higher than those in the control group (*p* < 0.05). The results showed that, compared with the control group, protease could promote nutrient absorption, improve small intestine morphology and enhance digestive enzyme activity in weaned piglets. The suitable addition amount was 150–300 mg/kg in the present study.

## 1. Introduction

Soybean is usually the main protein source in piglet diets [1]. Soybeans are typically high in antinutritional factors (ANF), such as trypsin inhibitors, soybean antigenic proteins, and soybean lectins, which reduce protein utilization by inhibiting digestive enzymes in the animal’s body, potentially leading to poor digestion, poor nutrition, and other effects [2]. These ANFs can be reduced by heat treatment, fermentation processes, and enzyme additions to improve protein utilization in pigs [2]. The results of many studies have shown that proteases can blunt or degrade anti-nutritional factors in feed to varying degrees [3,4,5]. 

Feed cost and environmental pollution have been affecting the development of modern pig farming. Soybean, the main protein source of pig diets, has led to increased feed costs due to its scarcity and high price. Incompletely digested proteins in pig diets are excreted in feces and urine and decomposed into ammonia, nitrate, and nitrite in the environment, causing nitrogen pollution [6]. Researches have shown that proteases can increase the digestion and absorption of protein materials and improve the digestive utilization of proteins and amino acids, thus, reducing the bad emissions of nitrogen [7,8,9,10]. Animals themselves can secrete a certain amount of endogenous proteases (pepsin, trypsin, chymotrypsin, etc.) to digest the nutrients in feeds, but there are still large amounts of nutrients in feeds that cannot be digested and utilized. In recent years, stand-alone proteases have been commercially available and have been added to pig diets in large quantities and have shown beneficial effects on nutrient digestibility and growth performance of pigs. The addition of proteases to diets significantly increased final body weight, endogenous protease activity, and intestinal villus height in weaned piglets and reduced feed-to-weight ratio, diarrheal index, and blood urea nitrogen, contributing to improved growth performance and nutrient digestibility and improved intestinal development and health in weaned piglets [11,12]. Furthermore, the addition of protease to the low-protein diet reduced the levels of leukocyte count, serum tumor necrosis factor-α (TNF-α), and transforming growth factor β (TGF-Β)-1 in weaned piglets [13]. Therefore, the addition of proteases to pig diets may be one of the strategies to improve nutrient utilization efficiency, reduce feed costs, and improve environmental pollution.

However, the application effects of proteases with different properties in diets are different. At present, most proteases used in the market are from the food industry, but their heat stability, degradation ability to substrates, and applicability to animal digestive physiology need to be improved. Recently, a new protease has been developed. The molecule is derived from the S2 family protease of thermophilic bacteria. Compared with the alkaline protease in the S8 family, which is widely used at present, it has higher heat resistance and acid resistance at the molecular level and has a wider range of enzyme cutting sites. It is widely used in various raw materials and forms a good complement with the endogenous protease. Studies have shown that the direct addition of this new protease can effectively improve the performance of poultry, increase the protein digestibility by 4–6%, increase the daily weight gain of broilers by 3–5%, improve the European index by more than 20, and greatly increase the economic benefits of broiler breeding [14]. Therefore, the effect of the new protease on pigs is worth exploring.

Until now, the effect of different proteases on pigs is still controversial [15,16,17]. Angel et al. [18] and Freitas et al. [19] showed that the beneficial effect of protease increased with the increase in protease dose in a linear [18] or quadratic [19] manner. The dose–response curve of any enzyme is inextricably linked to substrate availability and outcome indicators, and it is likely that the dose–response will vary with dietary composition, animal day age, etc. We were interested in conducting an evaluation of the effects of dietary supplementation with 0, 150, 300 and 600 mg/kg novel proteases on growth performance, diarrhea rate, nutrient digestibility, intestinal morphology, and health of weaned piglets.

## 2. Materials and Methods

All experimental procedures used in this study were approved by the Institutional Animal Care and Use Committee of Sichuan Agricultural University. The novel protease was a commercial product (10,000 U/g) from Jinan Bestzyme Bioengineering Co., Ltd., Jinan, China.

### 2.1. Experimental Animals, Design, Diets, and Housing

A total of 96 healthy [Duroc × (Landrace × Yorkshire)] weaned barrows aged 28 days were used in this study, with an average initial weight of 6.55 ± 0.83 kg. The piglets were randomly divided into 4 treatment groups with 6 replicates per treatment and 4 pigs per replicate according to a completely randomized block design. All piglets were divided into 4 treatment groups (6 replicates/group and 4 pigs/pen) in a completely randomized block design based on body weight. Piglets were fed the added protease levels included 0, 150, 300, or 600 mg/kg of diet, respectively. The feeding experiment lasted for 28 d. The basal diet was based on the National Research Council (NRC 2012) recommendations to meet or exceed nutritional requirements (Table 1). All pigs had free access to feed and water during the 28 d experiment. The ambient temperature was maintained at 24 ± 3 °C, and the relative humidity was controlled at around 50% ± 5%.

Feed intake in each column was recorded daily throughout the trial. After fasting for 12 h, weights were taken at the beginning, middle, and end of the experiment. The average daily feed intake (ADFI), average daily gain (ADG), and feed to gain (F:G) ratio were calculated. Pigs with dense or liquid feces are considered to have diarrhea. Diarrhea rate (%) = total number of diarrhea piglets per pen/(number of piglets per pen × number of days of the experiment phase) × 100 [20].

### 2.2. Sample Collection

Fecal samples were collected on days 25 to 28 of the experiment. Fresh feces were collected from each pen (4 piglets) immediately after defecation into their respective ziploc bags. Per 100 g feces uniform, 10 mL of 10%—H_2_SO_4_ solution needs adding to fix nitrogen manure.

At the end of the experiment, an average weight pig was selected from each pen for sample collection. Blood was collected from the anterior vena cava without anticoagulant and placed in a vacuum tube. After centrifugation (3500× *g*, 4 °C, 15 min), serum samples were collected and stored at −20 °C until analysis. The same piglets were anesthetized 2 h after the last feeding and samples were collected. The abdominal cavity was opened and the duodenum, jejunum, ileum, cecum, and colon were rapidly separated according to the anatomical structure. The abdominal cavity of the piglets was opened and the duodenum, jejunum, ileum, cecum, and colon were rapidly separated. Immediately thereafter, approximately 2 cm segments of intestinal tissue were separated from the proximal ends of duodenum, jejunum, and ileum, and care was taken to avoid extrusion during operation. Intestinal tissue after separating fixed in 4% paraformaldehyde solution in observing intestinal morphology. Villus height and crypt depth were measured. The chyme samples in each intestinal segment were collected into a sterile tube by carefully massaging the intestine, and the pH, amino acid digestibility, and digestive enzyme activities of chyme were determined. A section of duodenum, jejunum, and ileum from each piglet was washed with 0.9% ice-cold saline, and the intestinal mucosa was scraped with a sterile slide on a tray with ice cubes placed at the bottom. Digested digesta and mucosa samples were immediately frozen in liquid nitrogen and then stored at −80 °C for analysis.

### 2.3. Chemical Analyses

The fecal samples were dried for 72 h in a 65 °C oven, then crushed and ground through a 1 mm screen. The apparent total tract digestibility (ATTD) of DM, CP, GE, and organic matter (OM) was analyzed using Cr_2_O_3_ as an external indicator. Crude ash, DM, and chromium concentration was determined, as per our previous report [18]. Determination of CP using copper catalyst Kjeldahl method. With automatic adiabatic oxygen bomb calorimeter (Parr Instrument Co., Moline, IL, USA) measuring gross energy. Amino acid analyzer (L-8900, HITACHI. Tokyo, Japan) was used to measure the amino acids (AA) content. The apparent total tract digestibility (ATTD) of DM, OM, CP, and GE and the apparent ileal digestibility (AID) of AA were calculated using the chromium concentration in the diets and digesta. The calculation formula was shown below:

Digestibility (%) = (1 − A1/A × B/B1) × 100

where A = nutrient concentration in diet, A1 = nutrient concentration in feces or digesta, B = chromium concentration in diet, B1 = chromium concentration in feces or digesta.

### 2.4. Serum Metabolites

Serum concentrations of urea nitrogen (SUN), albumin (ALB), globulin (GLO), total protein (TP), alanine aminotransferase (ALT), and aspartate aminotransferase (AST) of piglets were measured using an automatic biochemical analyzer (7020, HI-TACHI, Japan). Measurement reference previously reported [21].

### 2.5. Digestive Enzyme Activity

The jejunal digesta was added with ice-cold saline at the ratio of weight (g): volume (mL) = 1:9. The digesta was mechanically homogenized in an ice-cold water bath, centrifuged at 2500× *g* for 10 min (4 °C), and the supernatant was taken for determination. The trypsin and chymotrypsin activities in the supernatant were then measured using commercial kits. The trypsin activity, chymotrypsin activity, and total protein content in the supernatant were determined by commercial kits. (Nanjing Jiancheng Bioengineering Institute, Nanjing, China, Sensitivity of trypsin kit: 10–8500 U/mL, Sensitivity of total protein kit: 0.2–1.3 mg/mL).

### 2.6. Determination of pH of Intestinal Digesta

The pH of the duodenal, jejunal, ileal, cecum, and colonic chyme was measured with a pH meter, according to the instructions, when collecting the intestinal chyme.

### 2.7. Intestinal Morphology

The fixed duodenum, jejunum, and ileum samples were removed from 4% paraformaldehyde solution for dressing. The samples were dehydrated (alcohol), transparent (xylene), and embedded (paraffin) and sectioned, then stained with hematoxylin and eosin (H&E), and sealed with neutral gum for histomorphological examination. Eclipse CI-L photo microscope (Nikon, Japan) was used to select the target area of tissues for 40× imaging. During imaging, tissues should be filled with the whole field of vision as far as possible to ensure the consistent background light of each photo. After the imaging was completed, image-Pro Plus 6.0 analysis software was used to measure the height of 5 intact villi in each section with mm as the standard unit. Five crypt depths were calculated and the average value was calculated.

### 2.8. Determination of Tight Junction Protein Gene Expression

Duodenal, jejunal, and ileal mucosa samples (approximately 0.1 g) at −80 °C were homogenized in 600 μL Trizol (TaKaRa, Dalian, China) reagent and then total RNA was extracted, according to the manufacturer’s instructions. Then, reverse transcription was performed using the PrimeScript RT kit and gDNA Eraser (Takara, Dalian, China), according to the manufacturer’s instructions. All samples were subjected to quantitative real-time PCR on the Q5 RealTime PCR detection system to analyze the gene expression level of zonula occludens-1 (ZO-1), occludin (OCLN), and claudin-1 (CLDN-1). β-actin was selected as the reference gene for transcription. Primers for specific genes (see Table 2 for primer sequences) were synthesized commercially by Shenggong Bioengineering (Shanghai, China). The specific experimental approach was adopted from our previous report [22].

### 2.9. Statistical Analysis

Experimental data were preprocessed by Microsoft Excel 2016 and then statistically analyzed by SPSS Statistics23 (IBM-SPSS Inc., Chicago, IL, USA). The data were tested for normality by Shapiro–Wilk test, and then analyzed by Linear Mixed Models program. Two-way ANOVA was used to analyze the effects of protease concentration and different time periods on the diarrhea rate of weaned piglets. Linear and quadratic models were established using the curve estimation procedure in regression analysis to further illustrate the concentration effect of protease in weaned piglets. Differences across treatments were determined by Duncan multiple comparisons. The results were presented as mean and standard errors (SEM). Significance was determined at *p* < 0.05 and 0.05 < *p* < 0.1 was considered a trend for difference.

## 3. Results

### 3.1. Growth Performance and Diarrhea Rate

The effect of protease on growth performance of weaned piglets is shown in Table 3. The addition of different concentrations of protease to the diet had no significant effect (*p* > 0.05) on ADFI, ADG, and F:G of weaned piglets.

The effects of protease on diarrhea rate of weaned piglets at different stages are shown in Table 4. The addition of different concentrations of protease at 1–14 d, 15–28 d, and 1–28 d after weaning had no significant effect on the diarrhea rate of piglets (*p* > 0.05).

### 3.2. Nutrient Digestibility

As shown in Table 5, compared with the control group, the supplementation of 150 mg/kg and 600 mg/kg protease had no effect on nutrient digestibility (*p* > 0.05), and the 300 mg/kg protease significantly increased the apparent digestibility of dry matter (DM), organic matter (OM), and gross energy (GE) (*p* < 0.05).

As shown in Table 6, compared with the control group, adding 600 mg/kg protease had no significant effect on amino acid digestibility (*p* > 0.05). For EAA, dietary protease supplementation significantly increased the apparent digestibility of Thr, Phe, and His (*p* < 0.05), and had a tendency to increase the apparent digestibility of Val, Leu, and Met (*p* > 0.05). For NEAA, adding the protease group significantly increased the apparent digestibility of Ser, Tyr, Pro, and Cys (*p* < 0.05).

### 3.3. Blood Metabolites

As shown in Table 7, compared with the control group, adding different concentrations of protease significantly increased serum globulin level (*p* < 0.05) and significantly decreased ALB/GLO (*p* < 0.05). The serum TP level of piglets increased with the increase in protease supplemental level (*p* > 0.05). Protease supplementation had no significant effect on serum albumin, ALT, AST, and SUN levels of piglets (*p* > 0.05).

### 3.4. Intestinal Morphology

**Figure 1:**
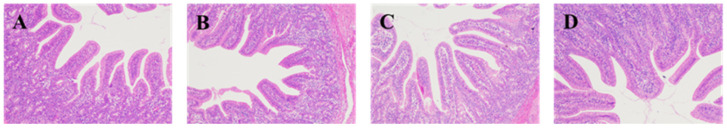
Effects of protease supplementation on duodenal morphology of weaned piglets: (**A**) the control group; (**B**) adding 150 mg/kg protease; (**C**) adding 300 mg/kg protease; (**D**) adding 300 mg/kg protease.

**Figure 2:**
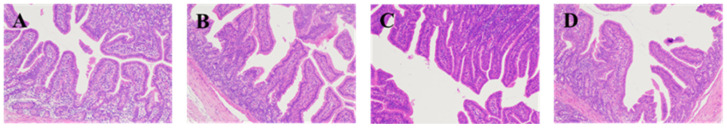
Effects of protease supplementation on jejunal morphology of weaned piglets: (**A**) the control group; (**B**) adding 150 mg/kg protease; (**C**) adding 300 mg/kg protease; (**D**) adding 300 mg/kg protease.

**Figure 3:**
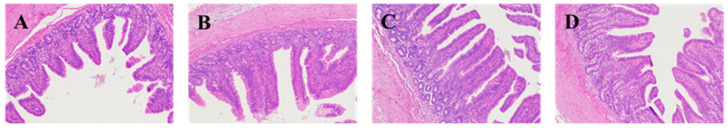
Effects of protease supplementation on ileum morphology of weaned piglets: (**A**) the control group; (**B**) adding 150 mg/kg protease; (**C**) adding 300 mg/kg protease; (**D**) adding 300 mg/kg protease.

As shown in Table 8 and Figure 1, Figure 2 and Figure 3, compared with the control group, dietary protease supplementation significantly increased villus height in duodenum of piglets (*p* < 0.05), and increased with the increase in protease concentration (*p* < 0.01, linear). The addition of 600 mg/kg protease significantly increased the crypt depth of duodenum (*p* < 0.05). Protease supplementation increased V:C ratio of duodenum of piglets to a certain extent and tended to increase with the increase in protease concentration (*p* > 0.05). Protease supplementation had no significant effects on villus height, crypt depth, and V:C ratio of jejunum and ileum of piglets (*p* > 0.05).

### 3.5. Intestinal Digesta pH

As shown in Table 9, compared with the control group, adding 300 mg/kg and 600 mg/kg protease into the diet significantly reduced pH of duodenal digesta (*p* < 0.05). Dietary protease supplementation had no significant effect on the pH value of other intestinal chyme (*p* > 0.05), but protease supplementation had a certain effect on pH value of other intestinal chyme.

### 3.6. Digestive Enzyme Activity

As shown in Table 10, compared with the control group, dietary protease supplementation significantly increased the trypsin and chymotrypsin activities in jejunal chyme of weaned piglets (*p* < 0.01), and the 300 mg/kg protease group had the highest digestive enzyme activities.

### 3.7. The mRNA Expression of Tight Junction Protein

As shown in Table 11, the addition of protease significantly upregulated the mRNA expression levels of ZO-1 (*p* < 0.01) and CLAUDIN-1 (*p* < 0.05) in the duodenal mucosa. In jejunum, the addition of protease significantly upregulated mRNA expression levels of ZO-1 (*p* < 0.05) and OCCLUDIN (*p* < 0.01). In addition, the addition of protease had no effect on the mRNA expression levels of tight junction proteins in ileal mucosa (*p* > 0.05). Interestingly, the protease showed a dose-dependent (*p* < 0.05, linear) upregulation of the gene expression of CLAUDIN-1, OCCLUDIN in the duodenum and jejunum of piglets, while the protease showed a dose-dependent (*p* < 0.05, quadratic) upregulation of the gene expression of ZO-1.

### 3.8. Optimum Addition Concentration of Novel Protease

As shown in Table 12, linear model and quadratic model for each significant effect were established by curve estimation procedure, and the best regression model and regression equation were obtained according to the significance coefficient (*p* < 0.05) of the model. The best regression model between serum Globulin, Albglo, and duodenum villi height of weaned piglets and chyme pH is the linear model. According to the regression equation, we found that adding 300 mg/kg protease to diet was the best concentration.

## 4. Discussion

Proteases can degrade feed proteins, but the digestive and secretory systems of young livestock and poultry are still in the developmental stage, resulting in a lack of endogenous proteases. Adding proteases to feed for young animals can promote digestion and absorption of nutrients and maintain the balance of normal intestinal flora [23,24,25]. The digestive system disorder of weaner piglets is caused by stress. Adding protease in the diet can supplement the deficiency of endogenous digestive enzyme secretion of weaner piglets, improve the digestion and absorption of nutrients, and reduce the diarrhea rate [11]. In this experiment, adding different doses of protease had no significant difference in diarrhea rate of piglets, but, compared with the control group, 300 and 600 mg/kg protease reduced the diarrhea rate of weaned piglets in different degrees at different stages. The statistically insignificant difference may be due to the fact that adding ZnO in the feed can reduce the diarrhea rate and interfere with the effect of protease.

Proteases hydrolyze proteins in rations into amino acids and small peptides, which are more amenable to intestinal digestion and absorption, thus, improving the digestibility of nutrients, such as proteins and amino acids, and also the digestibility of other nutrients in the feed. The addition of protease helps to neutralize anti-nutritional factors, such as protease inhibitors, thus, improving the digestibility of protein [26]. The results showed that the apparent digestibility of crude protein, dry matter, organic matter, energy, and amino acid of weaned piglets were affected by different levels of protease in diets, and there was a quadratic curve relationship between the apparent digestibility of crude protein, dry matter, organic matter, energy and amino acid of weaned piglets, and the 300 mg/kg protease group had the best effect. Feeding protease can improve protein utilization efficiency by complementing the deficiency of endogenous protease and acting synergistically with it [11]. In this experiment, the apparent ileal digestibility of some essential amino acids and non-essential amino acids was improved, which may be due to the insufficient secretion of endogenous protease after adding an appropriate amount of exogenous protease. However, when the added dose exceeds a certain level, it may negatively inhibit the secretion of endogenous protease and affect the activity of digestive enzymes [27,28]. Therefore, in this study, the high added level of protease did not play a role in improving the digestibility of nutrients. Zhou also confirmed that the addition of high dose neutral protease decreased the weight gain of piglets and the digestive enzyme activities in pancreas and jejunum of piglets [29]. The subsequent measurement of endogenous digestive enzymes in weaned piglets in this experiment was consistent with the previous report. These results indicate that high concentration of protease may have negative effects on nutrient digestibility and endogenous digestive enzymes of weaned piglets, compared with low concentrations of protease.

The majority of digestive enzymes are synthesized and secreted by the pancreas, but the secreted proenzyme is only able to function after it is activated in the intestine. Therefore, the activity of digestive enzymes in the digestive chyme can be used as an indicator to accurately reflect the changes in digestive enzymes. Exogenous protease can supplement the endogenous enzyme deficiency of the body, on the one hand, and stimulate the secretion of endogenous enzyme at the same time; therefore, the addition of protease to the diet will improve the digestive enzyme activity of the body and then improve the digestive utilization of nutrients and promote the improvement of growth performance. It has been reported that adding protease into the diet can improve the activity of endogenous enzymes in animals [11,30,31]. Xi added neutral protease into the basic diet of silk-hair black bone chicken, which all increased the activities of pepsin, trypsin, and total protein hydrolase to varying degrees [32]. Leng added protease to carp feed and found that protease activity in foregut tissue and chyme could be significantly increased when fish meal content was low [33]. Zhou added protease into the diet of weaned piglets, and the endogenous digestive enzyme activities of piglets were improved [29]. However, the digestive enzyme activities of pancreas and jejunum chyme were decreased when the high dose of protease was added. In this experiment, dietary protease supplementation significantly increased trypsin and chymotrypsin activities of jejunal chyme of weaned piglets, and the digestive enzyme activities were the highest when the protease supplementation was 300 mg/kg, which was consistent with previous studies.

Routine blood indicators can directly reflect the health status of animal organisms and also the deposition of nutrients in the animal’s body [34]. Total serum protein is composed of albumin and globulin and has the function of maintaining the normal osmotic pressure and pH of intravascular colloids and transporting a variety of metabolites [35]. The level of serum TP concentration can reflect the strength of protein anabolism in piglets. Serum globulin accounts for a large proportion of total serum protein and is mainly produced by the mononuclear macrophage system, when the level of circulating antibodies in the body increases, the amount of serum globulin also increases, it can protect the body by reacting immunologically with foreign specific antigens, the content reflects to some extent the immune level and physiological condition of the animals [36]. Albumin/globulin reflects the immune function of the spleen and is also an indicator of the immune level of the organism. The decrease in albumin/globulin indicates that the body elevates the level of specific immune response and enhances disease resistance. In this experiment, the addition of protease increased the serum globulin level and decreased the ALB/GLO level in piglets, and the serum TP level increased with the increase in the dose of protease, indicating that the addition of protease promoted protein synthesis and maintenance of blood osmolality.

Intestine is the main place for digestion and absorption of substances, and it is the first barrier in the body. The degree of intestinal development affects the absorption of nutrients in the intestine, which, in turn, affects the health of the body. The height of small intestinal villi is related to the number of cells, and the longer villi can expand the surface area of cells, which is beneficial to the absorption of nutrients; when the villi are short, the number of mature villi cells decreases, and the absorption capacity of nutrients is reduced. Due to the stress of weaning, piglets have some changes in the morphology of the intestine, with shorter villi and deeper crypt. How to recover the piglets’ intestine quickly after weaning is an important factor affecting their growth. In this experiment, adding protease in weaned piglets fodder significantly improves piglet duodenum villus height and V:C ratio has a tendency to increase with the increase in protease dose, while Zuo found that adding 200 or 300 mg/kg protease in fodder can improve the small intestinal villus height of weaned piglets and V:C ratio [11], the results are consistent with this. However, in this experiment, the addition of 600 mg/kg protease significantly increased duodenal crypt depth in weaned piglets, which may be related to the negative effect of high protease concentration on weaned piglets.

Changes in pH in the gut affect the number, as well as the type of intestinal microorganisms. Under normal conditions, beneficial microorganisms have an inhibitory effect on pathogenic microorganisms by metabolizing volatile fatty acids, lowering the pH of the gastrointestinal tract and inhibiting the growth and reproduction of pathogenic bacteria, but a high pH intestine is ideal for the proliferation of pathogenic microorganisms, which seriously affects intestinal health. In this experiment, the addition of different doses of protease significantly reduced the pH of duodenal chyme in weaned piglets, and the pH of chyme in jejunum, ileum and colon tended to decrease with increasing doses. The reduced gastrointestinal pH facilitated the secretion and activation of various proteases, and also promoted the growth of beneficial bacteria, inhibited the reproduction of pathogenic bacteria, and promoted intestinal health, which, in turn, improved production performance [37]. Ai and Han found that the addition of enzyme preparation reduced the pH of each intestinal segment of poultry [38], which was consistent with our study, indicating that the addition of protease can promote the intestinal health of weaned piglets.

Tight junctions play a crucial role in maintaining intestinal mucosal permeability and epithelial barrier integrity and promoting intestinal health, and they are mainly composed of transmembrane proteins occludin, claudins, etc., cytoplasmic proteins ZOs, and cytoskeletal structures [39,40]. Claudin, as the main functional protein of intercellular tight junctions, ensures the permeability of tight junctions [41]. Occludin functions are involved in intercellular adhesion, mobility, and permeability, and once mutated, reduced, or absent can cause increased permeability in the interstitial spaces of intestinal epithelial cells [42]. ZO-1 is associated with the maintenance and regulation of intestinal epithelial barrier and barrier function and is involved in important processes, such as cellular material transport and maintenance of epithelial polarity [43]. In this experiment, mRNA expression levels of ZO-1 and Claudin-1 in the duodenum and ZO-1 and occludin in the jejunum of weaned piglets were significantly up-regulated by the addition of protease, indicating that protease can up-regulate the mRNA expression levels of tight junction proteins, which is beneficial for improving the intestinal barrier. However, protease had no effect on the expression levels of related genes in the Ileum, which may be related to the differences in morphology, physiological function, and flora structure of different intestinal segments.

## 5. Conclusions

In conclusion, the present study demonstrated that the diet to add protease (300 mg/kg) can significantly improve the nutrient digestibility and endogenous digestive enzyme activities but did not change the growth performance. The intestinal morphology was improved and the expression of the tight junction protein was up-regulated. This study showed that for weaned piglets, the appropriate addition level of the novel protease was 300 mg/kg.

## Figures and Tables

**Table 1:** Ingredients and nutrient composition of the experimental diets (as-fed basis).

Item	Content
Ingredient, %	
Corn (CP 7.8%)	26.00
Corn, extruded (CP 7.8%)	31.00
Soybean meal (46%)	6.00
Soybean meal, extruded	6.00
Soy protein concentrate	5.00
Soybean meal, fermented	3.00
Rice, broken	7.00
Fish meal (CP 62.5%)	4.00
Whey powder (CP 3%)	5.00
Sucrose	2.00
Soybean oil	1.30
Choline chloride	0.10
NaCl	0.30
Vitamin premix ^a^	0.05
Mineral premix ^b^	0.40
Limestone	1.00
Dicalcium phosphate	0.51
L-Lysine-HCl (78%)	0.50
DL-Methionine (99%)	0.16
L-Threonine (98.5%)	0.16
L-Tryptophan (98.5%)	0.02
Citric acid	0.20
Benzoic acid	0.30
Nutrient levels ^c^	
CP, %	18.23
DE, MJ/kg	14.58
Ca, %	0.79
Total P, %	0.58
Available P, %	0.40
DLys, %	1.35
DMet, %	0.46
DMet + DCys, %	0.72
DThr, %	0.79
DTrp, %	0.22

^a^ Vitamin premix provides the following vitamins per kilogram of diet: vitamin A, 15,000 IU; vitamin D_3_, 5000 IU; vitamin E, 40 IU; vitamin K_3_, 5 mg; vitamin B_1_, 5 mg; vitamin B_2_, 12.5 mg; vitamin B_6_, 6 mg; vitamin B_12_, 0.06 mg; niacin, 50 mg; folic acid, 2.5 mg; biotin, 0.25 mg; pantothenic acid, 25 mg. ^b^ Mineral premix provided the following minerals per kilogram of complete diet: Fe 100 mg (FeSO_4_•H_2_O); Cu 6 mg (CuSO_4_•5H_2_O); Mn 4 mg (MnSO_4_•H_2_O); Zn 100 mg (ZnSO_4_•H_2_O), 1600 mg (ZnO); I 0.14 mg (KI); Se 0.30 mg (Na_2_SeO_3_). ^c^ Nutrient levels are calculated value.

**Table 2:** Primer sequences used for real-time PCR.

Gene	Accession No.	Primer Sequences (5′-3′)	Size, bp	AT, °C
ZO-1	XM_005659811.1	F: CAGCCCCCGTACATGGAGA	114	60
		R: GCGCAGACGGTGTTCATAGTT		
CLAUDIN-1	NM_001244539.1	F:TTTCCTCAATACAGGAGGGAAGC	214	60
		R: CCCTCTCCCCACATTCGAG		
OCCLUDIN	NM_001163647.2	F: CTACTCGTCCAACGGGAAAG	110	60
		R: ACGCCTCCAAGTTACCACTG		
β-actin	XM_003124280.5	F: TGGAACGGTGAAGGTGACAGC	177	60
		R: GCTTTTGGGAAGGCAGGGACT		

PCR = polymerase chain reaction; AT = annealing temperature.

**Table 3:** Effects of protease supplementation on performance in weaned piglets.

Item	Protease Concentration, mg/kg				SEM	*p*-Value		
	0	150	300	600		Treatment	Linear	Quadratic
Initial weight/kg	6.56	6.56	6.55	6.55	0.17			
Final weight/kg	13.90	13.65	13.39	14.13	0.16	0.87	0.88	0.47
1–14 d								
ADG (g)	176.31	177.08	180.30	192.35	0.45	0.45	0.11	0.99
ADFI (g)	314.29	308.24	310.80	331.47	0.72	0.72	0.35	0.67
F:G ratio	1.78	1.75	1.72	1.73	0.88	0.89	0.50	0.55
15–28 d								
ADG (g)	348.10	329.41	308.45	348.93	7.31	0.32	0.91	0.09
ADFI (g)	545.44	536.31	523.47	550.75	10.20	0.87	0.85	0.48
F:G ratio	1.58	1.63	1.69	1.58	0.02	0.09	0.84	<0.05
1–28 d								
ADG (g)	262.20	253.25	244.38	270.64	4.90	0.41	0.54	0.19
ADFI (g)	429.87	422.28	417.14	441.11	8.11	0.84	0.63	0.54
F:G ratio	1.64	1.67	1.70	1.63	0.01	0.37	0.76	0.12

ADFI = average daily feed intake; ADG = average daily gain; F:G ratio = feed-to-gain ratio.

**Table 4:** Effects of protease supplementation on diarrhea rate in weaned piglets.

Item	Protease Concentration, mg/kg				SEM	*p*-Value		
	0	150	300	600		Pr	Ph	Pr × Ph
1–14 d	1.79	3.57	1.49	2.08	1.00	0.12	0.53	0.99
15–28 d	1.89	2.38	0.89	0.60	0.44			
1–28 d	1.84	2.98	1.19	1.34	0.41			

Pr = Protease; Ph = Phase; Pr × Ph = Protease × Phase.

**Table 5:** Effects of protease supplementation on apparent total tract digestibility (ATTD) in weaned piglets.

Item	Protease Concentration, mg/kg				SEM	*p*-Value		
	0	150	300	600		Treatment	Linear	Quadratic
DM, %	84.67 ^b^	85.98 ^ab^	87.07 ^a^	84.64 ^b^	0.32	<0.01	0.82	<0.01
OM, %	87.65 ^b^	89.00 ^a^	89.80 ^a^	87.49 ^b^	0.29	<0.01	0.63	<0.01
CP, %	78.48	80.30	81.89	79.80	0.50	0.11	0.42	0.02
GE, %	85.18 ^b^	86.44 ^ab^	87.31 ^a^	85.15 ^ab^	0.32	0.03	0.81	<0.01

DM = dry matter; OM= organic matter; CP = crude protein; GE = gross energy. Within a row, mean values of different letter superscripts were significantly different (*p* < 0.05).

**Table 6:** Effects of protease supplementation on apparent ileal digestibility of amino acids in weaned piglets.

Item	Protease Concentration, mg/kg				SEM	*p*-Value		
	0	150	300	600		Treatment	Linear	Quadratic
EAA								
Arg, %	88.05	90.55	90.82	87.30	0.73	0.22	0.52	0.08
Thr, %	81.73 ^c^	85.79 ^ab^	86.83 ^a^	83.28 ^bc^	0.61	<0.01	0.65	<0.01
Val, %	81.09	84.55	85.54	82.41	0.69	0.08	0.73	0.01
Ile, %	82.72	85.58	86.37	83.48	0.70	0.22	0.91	0.04
Leu, %	85.52	88.72	89.16	86.40	0.58	0.06	0.89	<0.01
Phe, %	85.37 ^b^	88.55 ^ab^	89.07 ^a^	85.35 ^b^	0.62	0.04	0.69	<0.01
Lys, %	91.45	92.11	92.67	90.57	0.56	0.61	0.52	0.34
His, %	84.78 ^c^	88.68 ^ab^	90.76 ^a^	86.69 ^bc^	0.64	<0.01	0.46	<0.01
Met, %	91.20	92.19	94.03	91.84	0.41	0.08	0.58	0.02
NEAA								
Ser, %	83.42 ^b^	86.30 ^ab^	87.35 ^a^	83.32 ^b^	0.63	0.03	0.71	<0.01
Glu, %	85.95	86.49	87.75	84.11	0.81	0.48	0.41	0.33
Gly, %	73.00	75.42	77.25	68.16	1.91	0.38	0.31	0.28
Ala, %	83.92	85.43	86.95	84.16	0.58	0.23	0.96	0.05
Tyr, %	82.21 ^b^	87.64 ^a^	89.31 ^a^	83.59 ^b^	0.86	<0.01	0.88	<0.01
Asp, %	82.90	83.86	85.15	81.16	0.84	0.42	0.43	0.25
Pro, %	80.52 ^b^	84.90 ^a^	86.79 ^a^	83.79 ^ab^	0.78	0.02	0.23	<0.01
Cys, %	76.22 ^c^	79.93 ^bc^	84.87 ^a^	80.71 ^ab^	0.92	<0.01	0.08	<0.01

Within a row, mean values of different letter superscripts were significantly different (*p* < 0.05).

**Table 7:** Effects of protease supplementation on blood metabolites in weaned piglets.

Item	Protease Concentration, mg/kg				SEM	*p*-Value		
	0	150	300	600		Treatment	Linear	Quadratic
TP, g/L	37.03	38.62	39.42	40.92	0.64	0.19	0.03	0.32
Albumin, g/L	25.02	24.88	24.93	25.38	0.41	0.98	0.72	0.86
Globulin, g/L	12.02 ^b^	13.73 ^ab^	14.48 ^a^	15.53 ^a^	0.42	0.02	<0.01	0.06
ALB/GLO	2.13 ^a^	1.83 ^ab^	1.75 ^b^	1.64 ^b^	0.07	0.03	<0.01	0.05
ALT, U/L	70.26	63.59	49.87	59.40	3.41	0.20	0.23	0.06
AST, U/L	47.27	48.20	56.12	44.04	4.70	0.85	0.84	0.51
SUN, mmol/L	2.34	2.35	2.41	1.90	0.12	0.40	0.16	0.58

Within a row, mean values of different letter superscripts were significantly different (*p* < 0.05).

**Table 8:** Effects of protease supplementation on intestinal morphology in weaned piglets (μm).

Item	Protease Concentration, mg/kg				SEM	*p*-Value		
	0	150	300	600		Treatment	Linear	Quadratic
Duodenum								
Villus height	397.03 ^b^	403.71 ^b^	452.41 ^ab^	523.58 ^a^	16.34	0.01	<0.01	0.58
Crypt depth	147.19	135.25	151.30	165.35	4.37	0.10	<0.05	0.68
V:C ratio	2.75	2.98	3.04	3.18	0.10	0.48	0.13	0.38
Jejunum								
Villus height	424.26	425.37	438.48	448.68	10.88	0.86	0.39	0.83
Crypt depth	137.21	137.50	143.25	132.58	2.54	0.56	0.56	0.36
V:C ratio	3.10	3.11	3.09	3.41	0.10	0.64	0.25	0.78
Ileum								
Villus height	383.62	373.86	379.40	413.40	12.02	0.68	0.31	0.68
Crypt depth	131.11	147.38	131.77	143.92	3.81	0.32	0.47	0.80
V:C ratio	2.94	2.55	2.88	2.89	0.08	0.25	0.72	0.44

V:C ratio = the ratio of villus height to crypt depth. Within a row, mean values of different letter superscripts were significantly different (*p* < 0.05).

**Table 9:** Effects of protease supplementation on pH of intestinal chyme in weaned piglets.

Item	Protease Concentration, mg/kg				SEM	*p*-Value		
	0	150	300	600		Treatment	Linear	Quadratic
Duodenum	6.36 ^a^	5.66 ^ab^	5.12 ^b^	4.48 ^b^	0.23	0.02	<0.01	0.30
Jejunum	6.83	6.64	6.68	6.58	0.11	0.89	0.51	0.68
Ileum	7.24	6.90	6.94	6.82	0.08	0.26	0.08	0.13
Cecum	6.03	6.12	5.84	5.76	0.06	0.18	0.06	0.65
Colon	6.39	6.41	6.45	6.43	0.05	0.98	0.83	0.77

Within a row, mean values of different letter superscripts were significantly different (*p* < 0.05).

**Table 10:** Effects of protease supplementation on jejunum digestive enzyme activities in weaned piglets (U/mg prot).

Item	Protease Concentration, mg/kg				SEM	*p*-Value		
	0	150	300	600		Treatment	Linear	Quadratic
Chymotrypsin	33,060.90 ^b^	35,347.32 ^b^	50,136.69 ^a^	48,214.53 ^a^	2218.40	<0.01	<0.01	0.02
Trypsin	0.62 ^b^	0.86 ^b^	1.45 ^a^	1.32 ^a^	0.10	<0.01	<0.01	<0.01

Within a row, mean values of different letter superscripts were significantly different (*p* < 0.05).

**Table 11:** Effects of protease supplementation on mRNA expression levels of tight junction proteins in intestinal mucosa of weaned piglets.

Item	Protease Concentration, mg/kg				SEM	*p*-Value		
	0	150	300	600		Treatment	Linear	Quadratic
Duodenum								
ZO-1	1.00 ^b^	2.38 ^a^	2.94 ^a^	2.85 ^a^	0.20	<0.01	<0.01	<0.01
CLAUDIN-1	1.00 ^b^	1.33 ^b^	2.25 ^a^	2.12 ^a^	0.15	0.01	<0.01	<0.01
OCCLUDIN	1.00	1.21	1.56	1.96	0.16	0.18	0.02	0.43
Jejunum								
ZO-1	1.00 ^b^	1.24 ^b^	2.23 ^a^	2.31 ^a^	0.19	0.01	<0.01	0.07
CLAUDIN-1	1.00	1.05	1.54	1.54	0.11	0.12	0.04	0.20
OCCLUDIN	1.00 ^b^	1.24 ^b^	2.05 ^a^	2.20 ^a^	0.14	<0.01	<0.01	0.02
Ileum								
ZO-1	1.00	1.31	1.25	1.07	0.08	0.52	0.97	0.19
CLAUDIN-1	1.00	1.15	1.16	1.19	0.07	0.81	0.42	0.46
OCCLUDIN	1.00	1.15	1.10	1.03	0.08	0.92	0.95	0.57

Within a row, mean values of different letter superscripts were significantly different (*p* < 0.05).

**Table 12:** Appropriate regression equation between protease concentration and each significant effect.

Item		Regression Equation	R^2^	RSD
ATTD	DM	Y= −0.00002480X^2^ + 0.015X + 84.564	0.42	1.26
	OM	Y= −0.00002360X^2^ + 0.014X + 87.593	0.46	1.09
	GE	Y= −0.00002243X^2^ + 0.014X + 85.108	0.34	1.34
AID	Thr	Y= −0.00004983X^2^ + 0.032X + 81.824	0.42	2.28
	Phe	Y= −0.00004287X^2^ + 0.025X + 85.460	0.34	2.60
	His	Y= −0.00005447X^2^ + 0.036X + 84.699	0.52	2.27
	Ser	Y= −0.00004393X^2^ + 0.026X + 83.401	0.35	2.60
	Tyr	Y= −0.00007231X^2^ + 0.046X + 82.275	0.49	3.17
	Pro	Y= −0.00005190X^2^ + 0.037X + 80.536	0.37	3.16
	Cys	Y= −0.00006226X^2^ + 0.046X + 75.732	0.45	3.51
Blood metabolites	Globulin	Y= 0.006X + 12.493	0.36	1.69
	ALB/GLO	Y= −0.001X + 2.033	0.28	0.28
Enzyme activity	Chymotrypsin	Y= −0.077X^2^ + 75.475X + 31067.033	0.45	8131.17
	Trypsin	Y= −0.000004266X^2^ + 0.004X + 0.556	0.52	0.33
Duodenum	Villus height	Y= 0.224X + 385.469	0.40	63.33
	pH	Y= −0.003X + 6.206	0.38	0.90
	ZO-1	Y= −0.00001187X^2^ + 0.010X + 1.035	0.68	0.56
	CLAUDIN-1	Y= −0.00000582X^2^ + 0.006X + 0.898	0.50	0.53
Jejunum	ZO-1	Y= −0.000004268X^2^ + 0.005X + 0.888	0.36	0.78
	OCCLUDIN	Y= −0.000003429X^2^ + 0.004X + 0.913	0.50	0.52

Linear: Y = bX + C; Quadratic: Y = aX^2^ + bX + C; Y: Index, X: dietary protease concentration.

## Data Availability

The data are available on request from the corresponding author.
